# From early-life fluoxetine exposure to lifelong, sex-specific behavioral changes: decoding the dynamics of sensitive periods

**DOI:** 10.1038/s41380-025-03223-6

**Published:** 2025-09-09

**Authors:** Maria Teresa Gallo, Anaïs Virenque, Alessia Golinelli, Fabio Fumagalli, Eero Castrén, Paola Brivio, Francesca Calabrese

**Affiliations:** 1 Department of Pharmacological and Biomolecular Sciences “Rodolfo Paoletti”, Università degli Studi di Milano, Milan, Italy; 2 Neuroscience Center-HILIFE, University of Helsinki, Helsinki, Finland

**Keywords:** Neuroscience, Molecular biology

## Abstract

Early-life experiences shape neural networks, with heightened plasticity during the so-called “sensitive periods” (SP). SP are regulated by the maturation of GABAergic parvalbumin-positive (PV+) interneurons, which become enwrapped by perineuronal nets (PNNs) over time, modulating SP closure. Additionally, the opening and closing of SP are orchestrated by two distinct gene clusters known as “trigger” and “brake”. Interestingly alterations in SP markers have been identified in neuropsychiatric disorders, suggesting they may play a role in the emergence of these pathological conditions. Here, we investigate, in rats, whether the behavioral phenotypes observed in adults exposed to fluoxetine (FLX) during gestation or breastfeeding (until postnatal day 21) are due to alterations in SP dynamics. In line with the pathological-like adult phenotypes observed, the molecular results reveal a clear sex difference with significant changes in the density of PV+, in the proportion of PV+ cells surrounded by PNNs, as well as in the expression of trigger and brake genes across the lifespan, in the prefrontal cortex and dorsal hippocampus. In particular, we observed the strongest effect in the dentate gyrus (DG) of the dorsal hippocampus, with an anticipation in prenatal-FLX males and a delay in postnatal-FLX females of SP opening. We suggest that the molecular targets herein described may represent useful biomarkers to identify people with potentially increased vulnerability and, accordingly, we can hypothesize that strategies (pharmacological or not) aimed at correcting these abnormalities may be useful in preventing the pathological manifestation.

## Introduction

Brain development follows a timed sequence of processes, in which temporal accuracy is crucial. Any deviation from this sequence in the maturational trajectory can impair brain formation and function and, therefore, may set the stage for pathological conditions that often do not manifest until later in life.

In early life, ongoing interaction with the environment shapes and refines neural networks [1]. Repeatedly stimulated circuits are strengthened, while unused ones are pruned. Brain plasticity, i.e. the ability to adjust in response to experience, is heightened during the so-called “sensitive periods” (SP), which are specific windows of development that affect differently brain regions.

In this field, the primary visual cortex has been the premier model to study SP for years, and this has allowed researchers to demonstrate that sensitivity to monocular deprivation causes amblyopia only during the SP. Moreover, the visual cortex and brain plasticity at large are crucially maintained by a correct balance between excitation and inhibition [2, 3].

Among the regulators of SP, the maturation of GABA-ergic interneurons, i.e., the main inhibitory interneurons of the brain, particularly fast-spiking parvalbumin-expressing (PV+) basket cells, occurs with different timing across the brain regions thus contributing to the sequential onset of SP [4]. Changes in this interneuron subpopulation have been linked to neuropsychiatric disorders [5], with a reduction in PV+ interneurons found in rodent models of depression [6–9] and schizophrenia [10].

Important regulators of PV+ interneuronal plasticity and SP are the perineuronal nets (PNNs), i.e., specialized extracellular matrix surrounding the soma, and the proximal dendrites of PV+ interneurons [11, 12]. PNNs are known to mediate the closure of sensitive periods by stabilizing synapses and preventing further synaptogenesis [3, 13, 14]. Additionally, PNNs specifically regulate PV+ interneuron activity and excitability [15–17], and their enzymatic degradation disrupts excitatory input to fast-spiking PV+ neurons [18].

The opening and closing of SP seem to be controlled by two distinct clusters of genes referred to as “trigger” and “brake” genes [4, 19–23]. Trigger genes play a crucial role in initiating and accelerating the opening of SP by promoting PV+ cell maturation and modulating the balance between excitatory and inhibitory circuit activity. Conversely, brake genes regulate the formation of PNNs, thereby modulating the closure of sensitive period plasticity [23].

Interestingly, in recent years, it has been demonstrated that the administration of antidepressants, including fluoxetine (FLX), during adulthood induces changes in neuroplastic mechanisms [24]. Specifically, these compounds, by binding directly to the neuronal receptor tyrosine kinase 2 (TRKB), allosterically promote BDNF signaling [25, 26] and consequently improve neuronal plasticity. Accordingly, it has been shown that, in adult mice, antidepressants reduce cortical inhibition and reactivate a juvenile-like plasticity (iPlasticity) through the activation of TRKB receptors [27, 28].

On the contrary, in line with previous observations [29–36], we have recently demonstrated that perinatal FLX exposure, i.e. during gestation (prenatal) or breastfeeding (postnatal) has negative consequences since it induces psychiatric-like phenotypes in adult, but not adolescent rats [37].

Furthermore, prenatal administration of FLX altered the stress response in adolescence [38], suggesting that the changes induced by early manipulation are “subthreshold” and lead to a manifest pathological phenotype only after exposure to further stimuli.

Given the latency between the end of the pharmacological manipulation and the manifestation of the behavioral changes, we hypothesized that FLX affects the finely tuned processes of brain development by causing alterations in the dynamics of SP that occur prior to the emergence of pathological-like symptoms, predisposing subjects to disease development.

In line with this reasoning, here we investigate possible alterations in SP dynamics, using two related but independent approaches, i.e., by measuring (1) PV+ interneurons, PNNs formation around PV+ interneurons and (2) the expression of several trigger and brake genes.

## Material and methods

### Animals

Thirty-eight adult Wistar rats (19 females and 19 males, from Charles River, Germany) were introduced into the animal facility one month before mating. Each female was paired with a male and timed pregnancies were recorded upon detection of a vaginal plug designated as gestational day (GD) 0. Following plug detection, male rats were returned to their original cages, while females were individually housed and provided with nesting material. At the weaning (postnatal day (PND) 21) pups were separated by sex and placed in groups of 2–4 animals per cage. We employed 151 animals that were divided into different experimental groups (Supplementary Table 1).

Animals had *ad libitum* access to food and water, and the environment maintained a consistent 12-h light/dark cycle, with a temperature of 22 ± 2 °C and humidity at 50 ± 5%.

All procedures in this study were conducted in accordance with authorization n° 472/2021-PR approved by the Italian Health Ministry in line with the Italian legislation in animal experimentation (DL 26/2014) and conformed to the European Communities Council Directive of September 2010 (2010/63/EU). All efforts were made to minimize animal suffering and to reduce the number of animals used.

### Experimental paradigm and drug administration

**Fig. 1:**
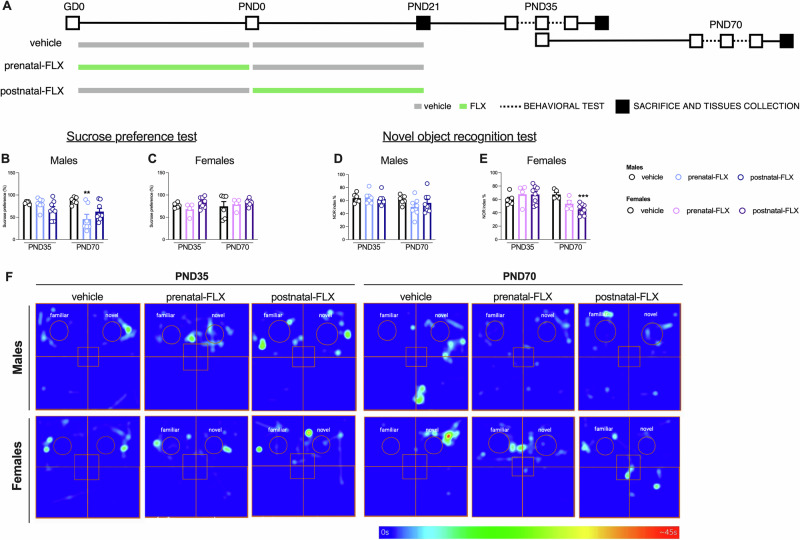
Schematic representation of the experimental paradigm and behavioral results. **A**: Male and female rats were exposed to fluoxetine (FLX) or vehicle (water) during gestation (prenatal-FLX) or breastfeeding (postnatal-FLX) and sacrificed at PND21, 35, or 70. Sucrose preference test (**B,** **C**) and novel object recognition test (**D,** **E**) were conducted during adolescence and adulthood. **F**: heatmaps show the animals’ head position for the entire duration of the test phases of the NOR, and use the scale shown below. Data are expressed as mean ± SEM of independent measures. ^**^p < 0.01, ^***^p < 0.001 vs vehicle at the same age (one-way ANOVA with Tukey’s multiple comparisons test). GD gestational day, PND postnatal day.

After mating, dams were randomly assigned to each experimental group. Specifically, 6 dams were exposed to the selective serotonin reuptake inhibitor fluoxetine (FLX) from GD0 to PND 0 (prenatal-FLX), 6 dams received FLX during breastfeeding (from PND0 to PND21; postnatal-FLX) and 7 dams were assigned to the control group assuming water along the whole experiment (vehicle) (Fig. 1).

As previously described [37, 38], dams assumed 15/mg/day of FLX dissolved in the drinking water. We tracked water intake before starting FLX administration to determine the proper dilution and considered an excess of solution to accommodate the daily water intake of the animals. During the exposure period, the solution was prepared daily to adjust the drug concentration based on the dams’ consumption and body weight.

### Behavioral tests

As represented in Fig. 1A, the behavioral tests were performed on the male and female offspring during adolescence (PND35) and adulthood (PND70). We first conducted the sucrose preference test (SPT) and then the novel object recognition (NOR) test. This specific order was carefully selected to progress from the least to the most invasive, reducing the risk of prior test affecting subsequent responses. Behavioral assessments were performed by an experimenter who was blinded to the animals’ experimental groups.

#### Sucrose preference test

As described previously [38], the animals were habituated to drinking water from two bottles in their home cages for three days before the test. Following this, all animals underwent a period of 15-h of food and water deprivation, after which they were housed individually. During the test, they were provided with one bottle containing a 1% sucrose solution and another containing water for 1 h. The position of the sucrose bottle was alternated to avoid side bias. The sucrose preference was calculated as [(ml of sucrose solution drunk)/(total ml of fluids drunk)] × 100.

#### Novel object recognition test

The test was carried out in a non-transparent squared open field (50 × 50 × 40 cm), and the test protocol [39] consisted of three stages: a five-minute training phase (two identical plastic bottles), one-hour inter-trial interval in the home cage, and a five-minute test phase (one plastic bottle and one tin can). Automatic assessment (ANY-maze software, Ireland) (Fig. 1F) was averaged with manual scoring of the time spent exploring the familiar object and the novel one. The NOR index was calculated by applying the following formula: [(time exploring the novel object/time exploring both the novel and familiar objects)] × 100.

### Tissues collection

Rats were decapitated at weaning, during adolescence, or in adulthood. We collected the whole brains of adolescents (3 rats per group) and fixed them by immersion in 4% PFA for 48 h at 4 °C. The brains were then cryoprotected in a solution of 30% sucrose in 1X PBS for 48 h at 4 °C, flash frozen, and sectioned with a cryostat into 40-μm-thick sections. The sections were collected with a fine brush, placed in a cryoprotectant solution, and stored at −20 °C for later immunohistochemical analyses.

At PND21, the frontal lobe and hippocampus were dissected from the whole brain, while at PND35 and 70 the prefrontal cortex (PFC) and the dorsal hippocampus (dHip) were collected [40]. The brain regions were frozen on dry ice and stored at −80 °C for later gene and protein expression analyses.

### Immunohistochemistry

Free-floating sections, containing the PFC or the dHip, were processed for the double fluorescent immunohistochemical staining.

Sections were blocked with 5% normal donkey serum (NDS) and 0.3% Triton X-100 (Sigma-Aldrich) in 1X PBS for 1 h at room temperature. Sections were incubated for 48 h at 4 °C with a primary antibody cocktail of biotin-conjugated Wisteria floribunda agglutinin (WFA) 1:200 (Sigma-Aldrich, Italy) and guinea pig anti-parvalbumin 1:1 000 (Synaptic System, Göttingen, Germany), diluted in 1X PBS with 1% NDS and 0.3% Triton X-100. After three rounds of 15 min washings in 1X PBS, the sections were incubated for 2 h at room temperature with a secondary antibody cocktail composed of Streptavidin Alexa Fluor 633 conjugate 1:500 (ThermoFisher Scientific, Italy) and goat anti-guinea pig Alexa Fluor 488 1:400 (Jackson immune research, Ely, United Kingdom), in 1% NDS and 0.3% Triton X-100 in 1X PBS and protected from light. Sections were rinsed three times with 1X PBS and then incubated with DAPI (1:5 000) for 5 min at room temperature to label the nuclei. Finally, sections were washed with 1X PBS for 10 min and cover-slipped with Fluoromount Aqueous Mounting Medium (Sigma-Aldrich, Italy).

### Image acquisition and analysis

Quantitative evaluation of immunostaining was performed blind to the experimental groups. The following brain regions were analyzed: prelimbic (PrL) and infralimbic (IL) subregions of the PFC and the dentate gyrus (DG), the CA1 and the CA3 areas of the dHip. These brain regions were determined according to the rat brain atlas Paxinos and Watson, 2007. Specifically, for PrL and IL cortexes we considered plates 9–13, while for DG, CA1, and CA3 plates 55–67 of the atlas [41].

A confocal laser scanning microscopy was used to detect the localization of the neurons positive for parvalbumin (PV+), perineuronal nets (PNNs+), and both (PV+PNN+). Images were obtained with the confocal microscope Nikon AX (Nikon, Amstelveen, The Netherlands) equipped with a 10x objective lens for PrL and IL and 20x objective lens for CA1, CA3, and DG, and imaging Software NIS Elements AR version 5.42.06 (Nikon, Amstelveen, The Netherlands).

From each brain section, a *z*-stack consisting of 20 consecutive images, with a step size of 1μm, was obtained. 3 sections per PrL-IL cortexes/animal, and 6 sections per DG-CA1-CA3/ animal, as well as the negative control sections, were imaged using the same microscope and camera settings for all slides. The brain areas analyzed belonged to both hemispheres of the brain of n = 3/group (6 hemispheres/group).

Image processing was done using ImageJ software (National Institutes of Health, Bethesda, MA, USA). To count the number of cells, a cell with detectable PV immunoreactivity above the background level was considered PV+; a cell with the soma surrounded with PNNs was considered PNN +; a cell with the soma that was not fully surrounded by PNNs on at least one image in *z*-stack was not counted as PNN +.

All images in each *z*-stack were analyzed. We calculated the density of PV+ cells by dividing the number of PV+ cells by the area, assessed the intensity of PV+ cells based on the mean gray value of each section, and investigated the percentage of total PV+ cells surrounded by PNNs.

### RNA preparation and gene expression analysis by quantitative real-time PCR

Total RNA was isolated from the frozen brain tissues by single-step guanidinium isothiocyanate/phenol extraction using PureZol RNA isolation reagent (Bio-Rad Laboratories, Segrate, Italy) and quantified by spectrophotometric analysis as previously described [42]. Samples were treated with DNase (ThermoFisher Scientific) to avoid DNA contamination.

Real-time polymerase chain reaction (q-PCR) was performed to measure total *Bdnf, Bdnf* long 3′ UTR, *Bdnf* isoform IV, *Bdnf* isoform VI, *Bmal1*, *Clock, Gad65, Gad67, Nptx2, Ncan*, *Bcan*, *Hapln1*, *Sem3a* mRNA levels. Primer sequences used were purchased from Eurofins MWG-Operon (Ebersberg, Germany) and Life Technologies (Italy) (Supplementary Tables 2, 3). mRNA was analyzed by Taqman qRT-PCR instrument CFX Opus 384 real-time PCR system, Bio-Rad Laboratories S.r.l., Segrate MI, Italy) using the iScriptTM one-step RT-PCR kit for probes (Bio-Rad Laboratories, Segrate, Italy) (see [40] for details). Samples were run in 384 well formats in triplicate as multiplexed reactions with normalizing internal control *36b4*.

### Protein extraction and Western Blot analysis

Western blot analysis was performed to investigate VGLUT1 and VGAT protein levels. DHip was manually homogenized using a glass−glass potter in a pH 7.4 cold buffer containing 0.32 M sucrose, 0.1 mM EGTA, and 1 mM HEPES solution in the presence of proteases (Roche, Monza, Italy) and phosphatases (Merck Life Science S.r.l, Milano, Italy) inhibitors. Total homogenates were sonicated, and total protein content was measured according to the Bradford Protein Assay procedure (Bio-Rad Laboratories, S.r.l., Segrate, Italy), using bovine serum albumin as a calibration standard. Equal amounts of protein were run under reducing conditions on 10% SDS-polyacrylamide gels and then electrophoretically transferred onto nitrocellulose membranes (Bio-Rad Laboratories S.r.l, Segrate, Italy). The blots were blocked with 5% nonfat dry milk (Euroclone, Milano, Italy), incubated with the primary antibodies diluted in 5% nonfat dry milk [VGLUT1 (Cell Signaling #12503) 1:750 4 °C O/N; VGAT: (Genetex # GTX101908) 1:1000 4 °C O/N; β-actin (Sigma-Aldrich # A5441) 1:10000 RT 45 min], and then incubated with the appropriate secondary ones (anti-rabbit Cell Signaling # BK3450S 1:750 RT 1 h, anti-rabbit 1:2000 RT 1 h, antimouse Merck #A4416 1:10000 RT 45 min, respectively). Immunocomplexes were visualized by chemiluminescence using the Chemidoc MP imaging system (Bio-Rad Laboratories S.r.l., Segrate, Italy). Results were standardized using β-actin as the control protein, which was detected by evaluating the band density at 43 kDa.

### Statistical analysis

GraphPad Prism software version 10.0 (GraphPad Software Inc., CA, USA) was employed to analyze all the results.

Behavioral and immunohistochemical results were subjected to the one-way analysis of variance (ANOVA) followed by Tukey’s multiple comparisons test. Protein expression data were analyzed with the unpaired *t*-test. Gene expression results were analyzed using the two-way ANOVA, with prenatal-FLX or postnatal-FLX and age as independent factors, followed by Tukey’s multiple comparisons test. Statistical significance for all the tests was assumed for p < 0.05.

For complete details about statistical analyses, please see Supplementary Information (Supplementary Table 4–15).

## Results

### Perinatal FLX induces different behavioral changes based on sex and periods of exposure

As shown in Fig. 1, one-way ANOVA revealed no significant effects of perinatal pharmacological exposure during adolescence in the SPT (Fig. 1B, C) or in the NOR test (Fig. 1D, E) in either males (SPT: F_2,19_ = 2.248, p > 0.05; NOR: F_2,19_ = 0.1332, p > 0.05) or females (SPT: F_2,17_ = 2.843 p > 0.05; NOR: F_2;17_ = 0.7721, p > 0.05).

However, according to previous observations [37], in adulthood a significant sex-specific effect of FLX exposure emerged (SPT-males: F_2,20_ = 6.186, p < 0.01. NOR-females: F_2,16_ = 11.32, p < 0.01). Specifically, the Tukey’s multiple comparisons test indicated that prenatal-FLX exposure significantly reduced sucrose preference in males (−47.4%, p < 0.01 vs vehicle), index of an anhedonic-like phenotype. Meanwhile, postnatal-FLX induced a significant reduction in the NOR index % (−32.9%, p < 0.001 vs vehicle), indicating deficits in memory in females.

### Differential effects of perinatal FLX on PV+ and PNN+ cells by timing of exposure, sex and brain region

**Fig. 2:**
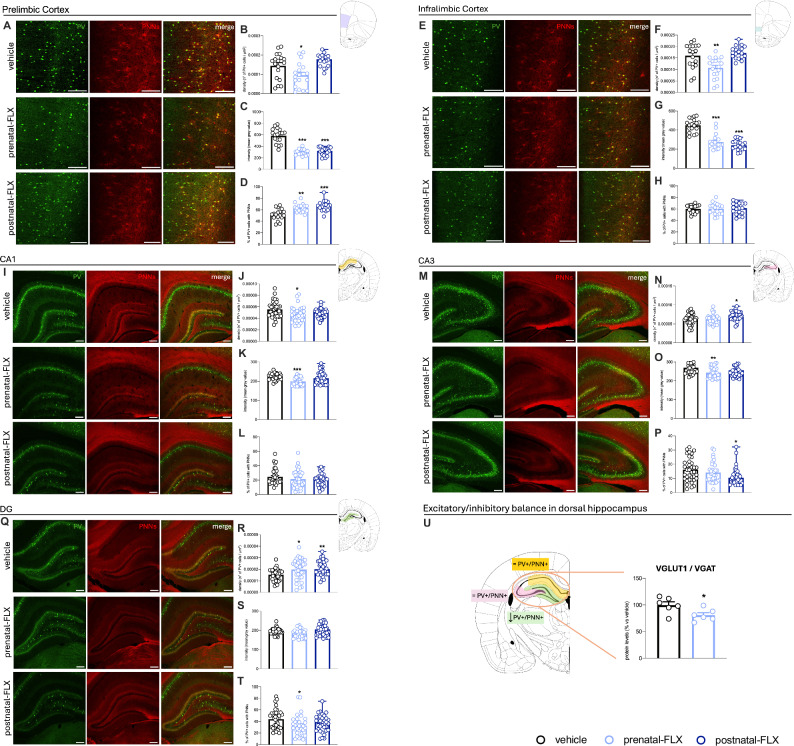
Quantification of PV+ cells, PNNs, and excitatory/inhibitory balance in adolescent male rats exposed to pre- or postnatal FLX. **A**–**T**: Quantification of parvalbumin-positive (PV+) cells and perineuronal nets (PNNs) in PrL (**A**–**D**), IL (**E**–**H**), CA1 (**I**–**L**), CA3 (**M**–**P**), DG (**Q**–**T**) of adolescent male rats exposed to pre- or postnatal-FLX. Representative images and quantification of PV+ (green) and PNN+ (red) and their merge to evaluate the percentage of PV+ cells surrounded by PNNs. Data are expressed as the mean ± SEM; ^*^p < 0.05, ^**^p < 0.01, ^***^p < 0.001 vs vehicle (one-way ANOVA with Tukey multiple comparisons test). Scale bar 200 μm. **U**: analysis of VGLUT1 and VGAT ratio in the dHip of adolescent male rats prenatally exposed to FLX. Data are expressed as the mean ± SEM; ^*^p < 0.05 (unpaired *t*-test vs vehicle).

In PrL of male rats, we observed that early exposure to FLX significantly affected PV+ cell density, reflecting the number of PV+ interneurons per area (F_2,53_ = 8.367, p < 0.001 vs vehicle, Fig. 2B), intensity, indicating the amount of PV protein (F_2,52_ = 57.34, p < 0.001. Figure 2C), and the percentage of PV+ cells surrounded by PNNs (F_2,51_ = 13.98, p < 0.001. Figure 2D).

In particular, prenatal-FLX led to a decreased density (−33.9%, p < 0.05 vs vehicle) and intensity (−48.9%, p < 0.001 vs vehicle) of PV+ cells, while the percentage of PV+ cells surrounded by PNNs was significantly increased (+21.9%, p < 0.01 vs vehicle). Similarly, postnatal-FLX reduced intensity (−46.5%, p < 0.001 vs vehicle) and increased the percentage of PV+/PNN (+29.0%, p < 0.001 vs vehicle).

Differently, in IL, FLX affected PV+ density (F_2,53_ = 11.72, p < 0.001 vs vehicle) and intensity (F_2,53_ = 51.44, p < 0.001. Figure 2G) with both the measures being reduced by the prenatal administration (density: −32.8%, p < 0.01 vs vehicle; intensity: −38.9%, p < 0.001 vs vehicle), while in the postnatal-FLX we found a selective reduction of PV+ intensity (−46.0%, p < 0.001 vs vehicle).

With respect to dHip (Fig. 2 panels I-T), one-way ANOVA revealed a significant effect of FLX on both the density and intensity of PV+ interneurons in CA1 (Density: F_2,101_ = 4.239, p < 0.05. Intensity: F_2,101_ = 10.90, p < 0.001. Figure 2J, K respectively) while in CA3 (Density: F_2,107_ = 5.576, p < 0.01. Intensity: F_2,106_ = 7.550, p < 0.001. Percentage of PV+ cells surrounded by PNNs: F_2,104_ = 3.605, p < 0.05. Figure 2N, O, P) and DG (Density: F_2,104_ = 6.908, p < 0.01. Intensity: F_2,103_ = 5.938, p < 0.01. Percentage of PV+ cells surrounded by PNNs: F_2,103_ = 4.171, p < 0.05. Figure 2R, S, T) all the parameters measured were affected.

In details, prenatal manipulation led to a decreased density in CA1 (−16.8%, p < 0.05 vs vehicle) and intensity of PV+ cells in both CA1 and CA3 (−12.2%, p < 0.001 vs vehicle; −10.0%, p < 0.01 vs vehicle, respectively), while in DG density was increased (+29.9%, p < 0.05 vs vehicle) and the percentage of PV+ cells surrounded by PNNs was reduced (−25.7%, p < 0.05 vs vehicle).

Finally, in the postnatal-FLX we observed an increased density of PV+ cells in CA3 and DG (+12.4%, p < 0.05 vs vehicle; +38.4%, p < 0.01 vs vehicle, respectively) and a reduction of the percentage of PV+/PNN (−28.4%, p < 0.05 vs vehicle, respectively) in the CA3.

In line, as represented in Fig. 2U, we found a lower VGLUT1/VGAT ratio in the dHip of the prenatal-FLX group compared with the vehicle (−19%, p < 0.05, Unpaired *t*-test).

**Fig. 3:**
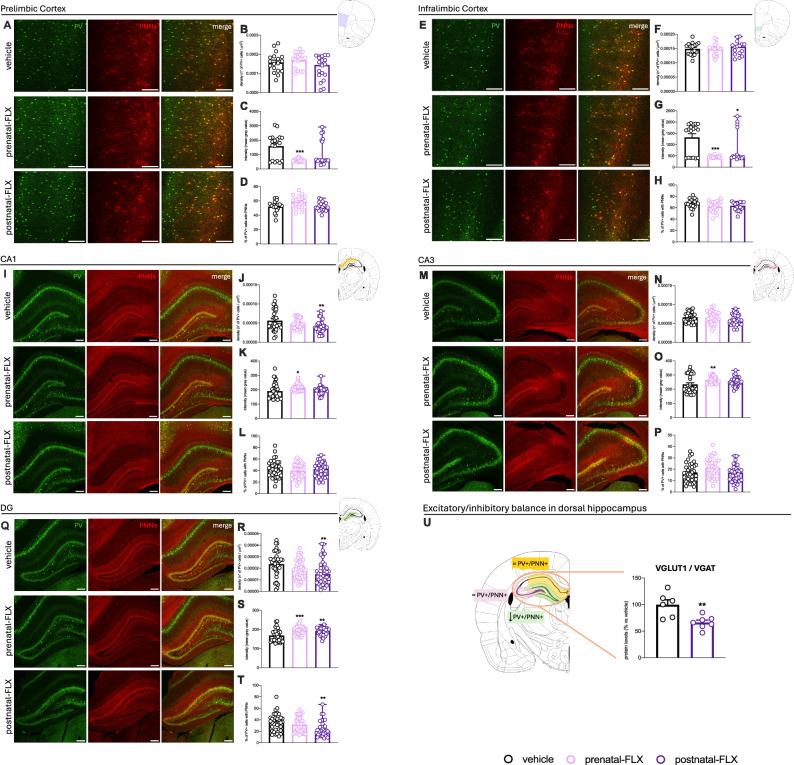
Quantification of PV+ cells, PNNs, and excitatory/inhibitory balance in adolescent female rats exposed to pre- or postnatal FLX. **A**–**T**: Quantification of parvalbumin-positive (PV+) cells and perineuronal nets (PNNs) in PrL (**A**–**D**), IL (**E**–**H**), CA1 (**I**–**L**), CA3 (**M**–**P**), DG (**Q**–**T**) of adolescent female rats exposed to pre- or postnatal-FLX. Representative images and quantification of PV+ (green) and PNN+ (red) and their merge to evaluate the percentage of PV+ cells surrounded by PNNs. Data are expressed as the mean ± SEM; ^*^p < 0.05, ^**^p < 0.01, ^***^p < 0.001 vs vehicle (one-way ANOVA with Tukey multiple comparisons test). Scale bar 200 μm. **U**: analysis of VGLUT1 and VGAT ratio in the dHip of adolescent female rats postnatally exposed to FLX. Data are expressed as the mean ± SEM; ^**^p < 0.01 (unpaired *t*-test vs vehicle).

As shown in Fig. 3B, F, one-way ANOVA analyses did not reveal any statistically significant effect of FLX on the density of PV+ cells in PrL (F_2,53_ = 3.154, p > 0.05) or IL (F_2,50_ = 0.113, p > 0.05) subregions in female rats.

In contrast, FLX significantly affected PV+ cell intensity in both prefrontal subregions (PrL: F_2,53_ = 8.117, p < 0.001. IL: F_2,51_ = 11.07, p < 0.001. Figure 3C and G respectively). Specifically, Tukey’s multiple comparisons test highlighted that prenatal-FLX led to decreased intensity in both PrL (−61.5%, p < 0.001 vs vehicle) and IL (−65.5%, p < 0.001 vs vehicle), while the postnatal manipulation selectively reduced intensity in the IL (−37.1%, p < 0.05 vs vehicle).

In the dHip, we observed a significant effect of FLX on PV+ cell density in CA1 (F_2,101_ = 4.727, p < 0.05. Figure 3J) and in DG (F_2,106_ = 4.470, p < 0.05. Figure 3R), but not in CA3 (F_2,106_ = 2.217, p > 0.05. Figure 3N). In contrast, PV+ cell intensity (Fig. 3K, O, S) was significantly affected by the pharmacological exposure across all hippocampal subregions analyzed (CA1: F_2,104_ = 4.274, p < 0.05. CA3: F_2,106_ = 5.055, p < 0.01. DG: F_2,106_ = 12.60, p < 0.001). In particular, the post hoc analysis revealed a reduced PV+ cell density in CA1 (−33.0%, p < 0.01 vs vehicle) and DG (−30.4%, p < 0.01 vs vehicle) in the postnatal-FLX group. Conversely, the PV+ intensity was selectively increased in the prenatal-FLX group in CA1 (+13.3%, p < 0.05 vs vehicle) and CA3 (+13.3%, p < 0.01 vs vehicle), while in DG, both prenatal-FLX (+16.9%, p < 0.001 vs vehicle) and postnatal-FLX (+12.6%, p < 0.01 vs vehicle) showed elevated intensity too.

Additionally, analysis of PV+ cells surrounded by PNNs revealed a statistically significant effect of FLX in CA3 (F_2,106_ = 4.227, p < 0.05. Figure 3P) and DG (F_2,104_ = 5.589, p < 0.01. Figure 3T) but not in CA1 (F_2,105_ = 0.774, p > 0.05. Figure 3L). Tukey’s multiple comparisons test indicated a decreased percentage of PV+PNN+ cells in the DG of the postnatal-FLX group (−31.4%, p < 0.01 vs vehicle).

Consistently, as represented in Fig. 3U, we found a lower VGLUT1/VGAT ratio in the dHip of the postnatal-FLX group compared with the vehicle (−34%, p < 0.01, Unpaired *t*-test).

### Prenatal-FLX in males alters trigger and brake gene expression profiles in the PFC and dHip across development

**Fig. 4:**
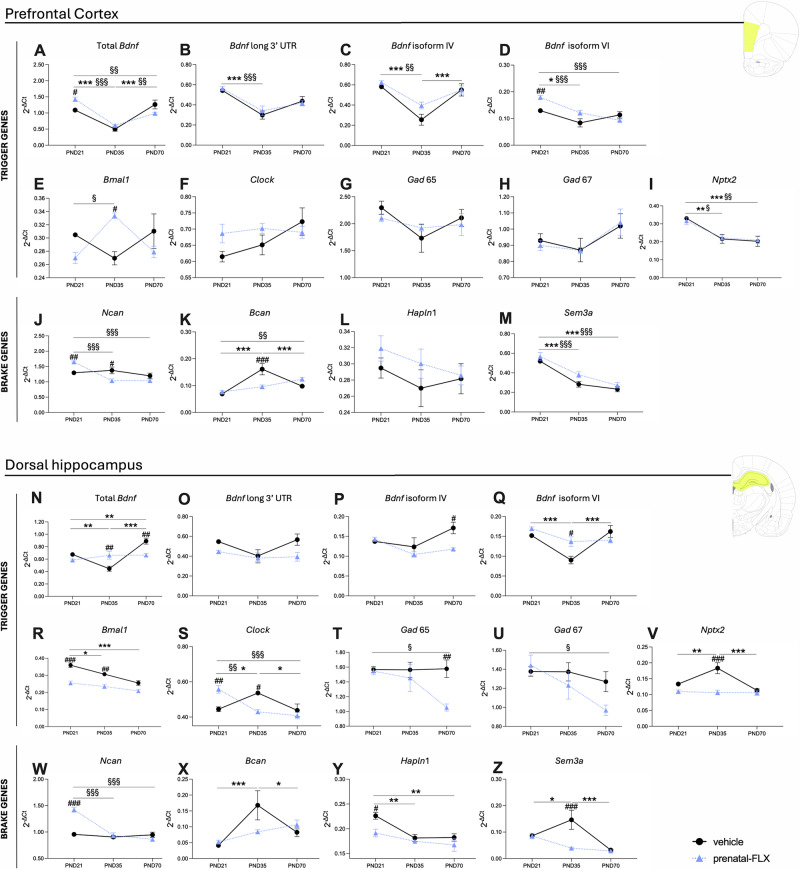
Analysis of trigger and brake genes of sensitive periods in the prefrontal cortex and dorsal hippocampus of male rats exposed to prenatal FLX. Panels **A**–**I** show trigger gene expression in the prefrontal cortex, and panels **J**–**M** show brake gene expression in the prefrontal cortex. Panels **N**–**V** represent trigger gene expression in the dorsal hippocampus, while panels W–Z show brake gene expression in the dorsal hippocampus. Data are expressed as mean ± SEM of independent measures. ^*^p < 0.05, ^**^p < 0.01, ^***^p < 0.001 between vehicle at different ages; ^§^p < 0.05, ^§§^p < 0.01, ^§§§^p < 0.001 between prenatal-FLX at different ages; ^#^p < 0.05, ^##^p < <0.01, ^###^p < 0.001 vs vehicle at the same age. (Two-way ANOVA with Tukey’s multiple comparisons test). PND postnatal day.

**Fig. 5:**
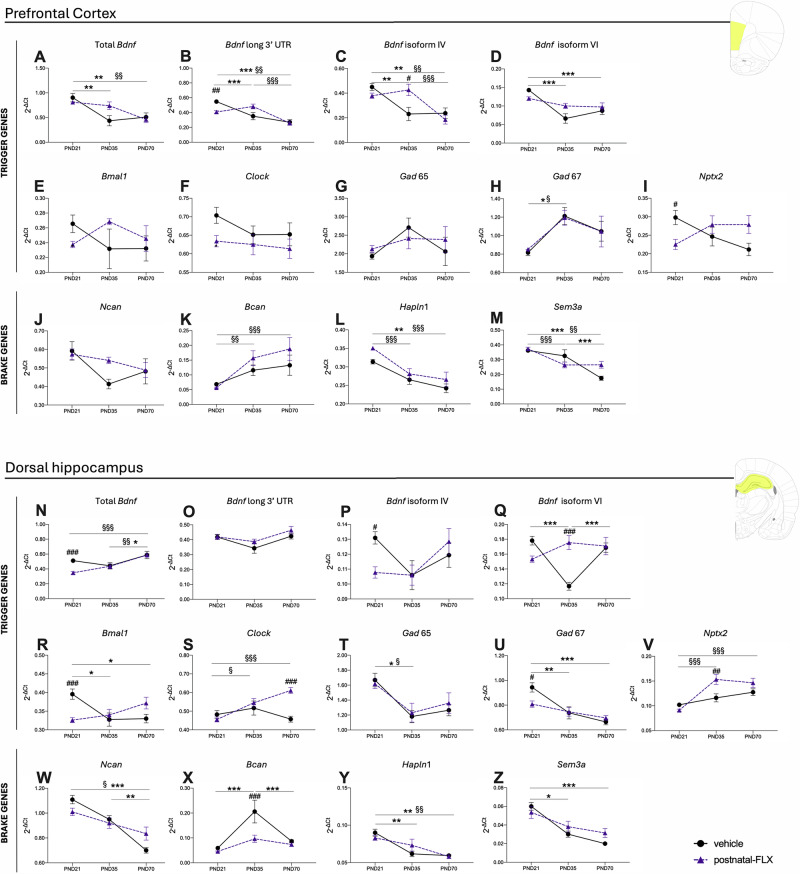
Analysis of trigger and brake genes of sensitive periods in the prefrontal cortex and the dorsal hippocampus of female rats exposed to postnatal  FLX. Panels **A**–**I** show trigger gene expression in the prefrontal cortex, and panels **J**–**M** show brake gene expression in the prefrontal cortex. Panels **N**–**V** represent trigger gene expression in the dorsal hippocampus, while panels **W**–**Z** show brake gene expression in the dorsal hippocampus. Data are expressed as mean ± SEM of independent measures. ^*^p < 0.05, ^**^p < 0.01, ^***^p < 0.001 between vehicle at different ages; ^§^p < 0.05, ^§§^p < 0.01, ^§§§^p < 0.001 between postnatal-FLX at different ages; ^#^p < 0.05, ^##^p < <0.01, ^###^p < 0.001 vs vehicle at the same age. (Two-way ANOVA with Tukey’s multiple comparisons test). PND postnatal day.

Based on the immunohistochemical results, to identify alterations in the dynamics of sensitive periods that could underpin the differential expression of PV+ interneurons and PNNs formation observed, we investigated the expression of several trigger (total *Bdnf*, *Bdnf* long 3′UTR, *Bdnf* isoform IV, *Bdnf* isoform VI, *Bmal1, Clock, Gad65, Gad67, Nptx2*) and brake genes (*Ncan, Bcan, Hapln1, Sem3a*) (Figs. 4, 5) in the group of rats exhibiting pathological-like phenotypes in adulthood (males exposed to prenatal-FLX and females exposed to postnatal-FLX).

In the PFC of males, we found that the prenatal-FLX group exhibited increased expression of total *Bdnf* and *Bdnf* isoform VI at PND21 (+12%, p < 0.05 and +40.5%, p < 0.01 vs vehicle, respectively) (Fig. 4A, D), whereas *Bmal1* mRNA levels were significantly upregulated by prenatal-FLX exposure specifically at PND35 levels (+23.8%, p < 0.05 vs vehicle).

Upon analyzing the brake genes, prenatal-FLX exposure led to an increase in *Ncan* expression at PND21 (+27.6%, p < 0.01 vs vehicle) and a decrease at PND35 (−22.7%, p < 0.05 vs vehicle. Figure 4J). At PND35 also *Bcan* expression levels (Fig. 4K) were decreased in the prenatal-FLX group (−40.6%, p < 0.001 vs vehicle).

In the dHip, we observed an upregulation of total *Bdnf* (Fig. 4N) and *Bdnf* isoform VI (Fig. 4Q) at PND35 (+48.8%, p < 0.01 vs vehicle; +51.5%, p < 0.05 vs vehicle, respectively) in the animals exposed to FLX probably due to the relatively stable expression of these factors in the prenatal-FLX group from PND21 to PND70, while the vehicle group showed a decrease from PND21 to PND35 followed by an increase from PND35 to PND70. Additionally, prenatal-FLX exposure led to a lower expression of total *Bdnf* (Fig. 4N) and *Bdnf* isoform IV (Fig. 4Q) at PND70 (−25.3%, p < 0.01 vs vehicle; −31.1%, p < 0.05 vs vehicle, respectively).

For *Bmal1* (Fig. 4R), we observed a significant decrease at both PND21 (−29.1%, p < 0.001 vs vehicle) and PND35 (−23.4%, p < 0.01 vs vehicle) in the prenatal-FLX group.

Differently, for the *Clock* gene (Fig. 4S), the post hoc analysis revealed a physiological increase from PND21 to PND35 (+20.7%, p < 0.05 vs vehicle-PND21) in the vehicle group, whereas an opposite effect was observed following prenatal-FLX exposure with an increased expression at PND21 (+25.4%, p < 0.01 vs vehicle) and reduced expression at PND35 (−19.9%, p < 0.05 vs vehicle).

*Gad65* expression was lower at PND70 (−33.3%, p < 0.01) in the prenatal-FLX group compared to vehicle group due to a downregulation observed from PND21 to PND70 (−31.9%, p < 0.05 vs prenatal-FLX-PND21) in this group, while animals exposed to vehicle showed stable mRNA levels across this period (Fig. 4T).

Additionally, *Nptx2* expression (Fig. 4V) was reduced in the prenatal-FLX group at PND35 (−41.9%, p < 0.001 vs vehicle), probably because of the perturbation of the physiological pattern of expression that is indeed relatively constant from PND21 to PND70 in the prenatal-FLX group, while the vehicle group exhibited an increase from PND21 to PND35 (+37.5%, p < 0.01 vs vehicle-PND21) and a decrease from PND35 to PND70 (−38.2%, p < 0.001 vs vehicle-PND35).

Moving to the brake genes, we observed higher expression of *Ncan* specifically at PND21 in the prenatal-FXL group (+48.8%, p < 0.001 vs vehicle) (Fig. 4W) whereas *Sem3a* expression (Fig. 4X) was increased in the vehicle group from PND21 to PND35 (+70.4%, p < 0.05 vs vehicle-PND21), but not in the prenatal-FLX group. Indeed, at PND35, *Sem3a* mRNA levels were decreased by prenatal-FLX (−73.2%, p < 0.001 vs vehicle).

Differently, the mRNA levels of *Hapln1* were decreased at PND21 (+15.4%, p < 0.05 vs vehicle) by the prenatal pharmacological manipulation (Fig. 4Y).

### Postnatal-FLX in females alters trigger and brake gene expression profiles in the PFC and dHip across development

In parallel with the analyses conducted in males, we also examined the expression of trigger and brake genes in females. In the PFC, postnatal-FLX exposure reduced the expression of *Bdnf* long 3′UTR at PND21 (−25.5%, p < 0.01 vs vehicle) (Fig. 5B) and increased *Bdnf* isoform IV expression at PND35 (+84.5%, p < 0.05 vs vehicle) (Fig. 5C).

Interestingly, the expression pattern of *Bdnf* isoform IV differed markedly between FLX-exposed and vehicle groups. Indeed, in the vehicle group, *Bdnf* isoform IV mRNA levels physiologically decreased between PND21 and PND35 (−48.6%, p < 0.01 vs vehicle-PND21) reaching a plateau, which is a typical developmental regulation. In contrast, in the postnatal-FLX group, expression remained stable between PND21 and PND35 and significantly decreased from PND35 to PND70 (−56.5%, p < 0.001 vs postnatal-FLX-PND35).

Moreover, *Nptx2* expression (Fig. 5I) was decreased at PND21 in the postnatal-FLX group (−24.4%, p < 0.05 vs vehicle).

In the dHip, the expression of total *Bdnf* (Fig. 5N) and *Bdnf* isoform IV (Fig. 5P) were lower at PND21 (−32.0%, p < 0.001 and −17.7%, p < 0.05 vs vehicle, respectively) in the postnatal-FLX group whereas *Bdnf* isoform VI (Fig. 5Q) displayed a higher expression at PND35 in the postnatal-FLX group (+50.2%, p < 0.001 vs vehicle).

Similarly, *Bmal1* and *Gad67* levels were significantly lower at PND21 in the postnatal-FLX group compared to vehicle (−17.6%, p < 0.001, −14.4%, p < 0.05 vs vehicle, respectively. Figure 5R, U), while *Nptx2* (Fig. 5V) and *Clock* (Fig. 5S) expression were higher at PND35 and PND70 (respectively) in the FLX-exposed animals (*Nptx2:* + 32.1%, p < 0.01 vs vehicle; *Clock:* + 33.6%, p < 0.001 vs vehicle).

Considering the brake genes, the multiple comparison tests revealed lower mRNA levels of *Bcan* at PND35 in the postnatal-FLX group compared with vehicle (−53.2%, p < 0.001 vs vehicle). This decrease was driven by an increase between PND21 and PND35 (+247.9%, p < 0.001 vs vehicle-PND21) in the vehicle group, followed by a subsequent decline at PND70 (−57.9%, p < 0.001 vs vehicle-PND35), whereas this pattern was absent in the postnatal-FLX group (Fig. 5X).

## Discussion

This study demonstrates that perinatal exposure to FLX significantly influences the maturation of the brain by altering the dynamics of sensitive periods (SP) within the prefrontal cortex (PFC) and dorsal hippocampus (dHip). Specifically, the findings presented herein reveal changes in the density of fast-spiking GABAergic parvalbumin-positive (PV+) interneurons, the proportion of perineuronal nets (PNNs) encapsulating PV+ cells during adolescence, and the expression of genes regulating the initiation (trigger) and termination (brake) of SP throughout the lifespan. Notably, similarly to the behavioral alterations observed in adulthood [37], these effects depend on the timing of FLX exposure, as well as on sex and on the brain region considered.

Firstly, we confirmed our previous findings at the behavioral level [37, 38] showing that pathological-like phenotypes induced by perinatal FLX administration become manifest in adulthood, with no observable alterations during adolescence. Male rats demonstrated greater sensitivity to prenatal FLX exposure and developed an anhedonic-like phenotype, while females were more adversely affected by postnatal FLX exposure, as shown by cognitive impairments. This is in line with evidence showing that the effect of psychoactive compound during development is sex dependent [43–46]. We believe that this notion is extremely relevant and must be taken into account when designing clinical trials for drug discovery in neurodevelopmental disorders [47].

Starting with males, we demonstrated that prenatal FLX exposure led to a reduced density and intensity of PV+ interneurons in the PFC and in the CA1 and CA3 subregions of the dHip during adolescence.

Moreover, in the PrL, we found an increased abundance of PV+ cells surrounded by PNNs, an indicator of stronger and/or faster stabilization of PV+, suggesting reduced plasticity compared to physiological conditions. This, in turn, suggests that prenatal FLX exposure anticipates the closure of the SP. In line with this, we found a reduced expression of *Ncan* and *Bcan* at PND35 due to prenatal-FLX. This result is interesting given the distinct roles of these two genes [48]. In fact, *Ncan* is involved in the development of the nervous system, and it is expressed predominantly in the early PNNs [48], while *Bcan* is critical for organizing the extracellular matrix and maintaining synaptic integrity and adaptability [48, 49]. These findings indicate that prenatal FLX also alters the composition and structure of the PNNs.

Unlike in the PrL, in the DG of the dHip, we observed increased PV density and a reduced percentage of PV+PNN+ cells, suggesting an anticipation in the opening of the SP.

In accordance with this hypothesis, we observed dysregulation of key processes governing SP dynamics. Specifically, at PND35, total *Bdnf* and *Bdnf* isoform VI were upregulated, while the expression of *Bcan* and *Sem3a* was reduced. These results are in line with evidence that, in visual cortex, the onset of the critical period can be accelerated by transgenic overexpression of BDNF [19].

Interestingly, given the protective role of PNN [14], it is possible to hypothesize that such changes could make neurons excessively plastic and vulnerable to negative stimuli [14].

Moreover, BCAN has been shown to play a key role in gating the function of PV+ interneurons, thereby enabling coordinated circuit responses to experience [15].

Together, the reduction of PNNs and Bcan could concur to the inefficient response to stress we previously observed in these animals at the same age [38].

**Fig. 6:**
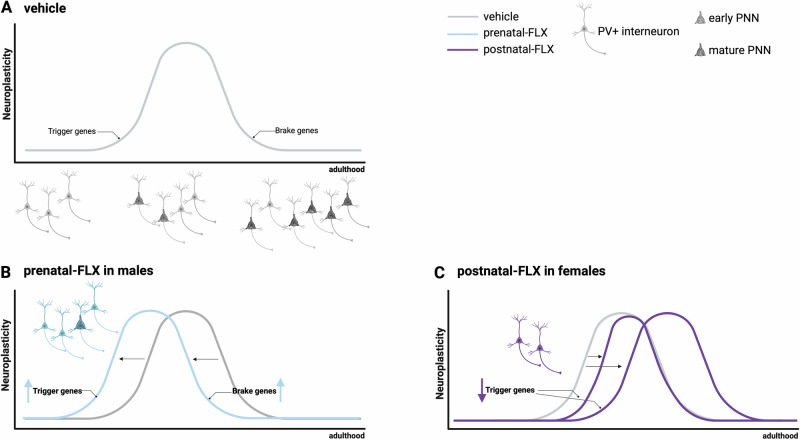
Neuroplasticity trajectory in the DG subfield of dHip of rats exposed to perinatal-FLX. Arrangement and development of parvalbumin positive (PV+) interneurons begin during early life and through development into adulthood the proportion of PV+ cells surrounded by perineuronal nets (PNNs) steadily increases as the brain reaches maturity. The opening of sensitive periods (SP) is regulated by the expression of the so-called “trigger genes” as well as the closure depending on “brake genes”. **A** shows the physiological situation (vehicle). **B** represents the anticipated opening and closure of SP induced by prenatal-FLX (light-blue) in male rats. **C** represents the delayed opening of the SP induced by postnatal FLX (purple) in female rats, with the possibility that closure occurs at the right time or is delayed.

Additionally, the reduced expression of total *Bdnf*, *Bdnf* isoform IV and *Gad65* at PND70, potential indicators of reduced neuroplasticity, suggests not only an anticipation of the opening of SP but also an anticipation of their closure (Fig. 6A, B).

Similarly to males, we observed region-specific effects in females, with changes especially in the DG subfield of the dHip. Notably, the DG is one of the most dynamic regions in the mammalian brain, known for its ability to generate adult-born principal neurons that integrate into existing neural circuitry [50, 51]. Indeed, notable changes were identified in the dHip, particularly within the DG subfield. Specifically, postnatal FLX exposure resulted in a reduction in PV+ cell density, an increase in PV+ intensity, and a lower percentage of PV+ cells surrounded by PNNs.

These findings indicate that postnatal manipulation delayed the maturation of PV+ interneurons as well as the opening of the SP. Typically, physiological development involves an increase in PV+ interneuron density and a concurrent decrease in PV protein levels within these cells together with an increase in PNNs formation [14, 52, 53]. Consistent with this, we observed reduced *Bcan* gene expression at PND35.

Moreover, considering that BCAN depletion in PV+ cells and transgenic *Bcan* mutations in mice have been linked to short-term memory defects [15], the herein shown reduction of *Bcan* might represent a contributing factor to the cognitive deficits previously observed in the dHip of adult females exposed to postnatal-FLX [37].

Further supporting this hypothesis, at PND21, the postnatal-FLX group exhibited reduced expression of several opening genes, including total *Bdnf*, *Bdnf* isoform IV, *Bmal1* and *Gad67*, indicating reduced neuroplasticity compared to vehicle group. These findings confirm a delay in the upregulation of these genes and, consequently, in the opening of the SP (Fig. 6A, C).

Finally, in the dHip of both male and female rats we found a lower ratio between the glutamate and GABA vesicular transporters, an indirect indicator of excitatory/inhibitory balance [54]. According with the literature showing that PNNs support PV+ neuron function by facilitating their high firing rate [15, 55, 56], this result is in line with the reduction of PV+/PNN cells we observed in the DG.

Altogether, our results demonstrate that perinatal FLX alters the dynamics of SP mainly in the DG of the dHip in a sex-dependent fashion with an anticipation in male rats exposed to prenatal-FLX and a delay in postnatal-FLX treated females, which are indeed specific for the groups that develop the pathological phenotypes at adulthood (anhedonia in prenatal-FLX males and cognitive deficits in postnatal-FLX females).

These effects depend upon a complex perturbation of the developing brain, presumably due to both the direct effect of FLX on neuroplasticity and the alteration of the serotonergic system.

Indeed, we previously demonstrated that FLX directly interacts with the TrkB receptor [24] and, in addition, the interplay between serotonin, neuroplasticity and the GABAergic system is very close and widely demonstrated (see [1, 57]).

These findings strengthen the evidence that any variation from the physiological developmental trajectory can impair brain plasticity and increase susceptibility to neuropsychiatric disorders in adulthood.

On this basis, the molecular targets described herein may represent, in the future, useful biomarkers to identify people with a potential increased vulnerability. Accordingly, we hypothesize that strategies (pharmacological or not) aimed at correcting these abnormalities may be useful in preventing the pathological manifestation.

## Supplementary information

Supplementary material

## Data Availability

All data generated or analysed during this study are included in this published article [and its supplementary information files]. If needed, other information is available on request from the authors.
