# Supplementary material for: From early-life fluoxetine exposure to lifelong, sex-specific behavioral changes: decoding the dynamics of sensitive periods
Source: Mol Psychiatry. 2025 Sep 9;31(3):1205–15. doi: 10.1038/s41380-025-03223-6 (PMC12916491; doi:10.1038/s41380-025-03223-6)
Supplement: Supplementary file 1 — Supplementary material [file 41380_2025_3223_MOESM1_ESM.docx]

| **EXPERIMENTAL GROUP** | **LITTER** | **N° MALES** | | | **N° FEMALES** | | |
| --- | --- | --- | --- | --- | --- | --- | --- |
|  |  | **PND21** | **PND35** | **PND70** | **PND21** | **PND35** | **PND70** |
| **vehicle** | **1** | 1 | 2 |  | 2 | 2 |  |
|  | **2** | 1 | 1 |  | 2 | 1 |  |
|  | **3** |  |  |  | 3 |  |  |
|  | **4** |  |  |  | 3 |  |  |
|  | **5** | 6 |  |  |  |  |  |
|  | **6** |  | 3 | 4 |  | 3 | 3 |
|  | **7** |  | 3 | 3 |  | 3 | 3 |
| **prenatal-FLX** | **8** | 2 | 2 |  | 2 | 2 |  |
|  | **9** | 2 | 1 |  |  | 1 |  |
|  | **10** | 1 |  |  |  |  |  |
|  | **11** | 2 |  |  | 6 |  |  |
|  | **12** |  | 2 | 2 |  | 1 | 1 |
|  | **13** |  | 5 | 5 |  | 3 | 3 |
| **postnatal-FLX** | **14** | 4 |  |  | 5 |  |  |
|  | **15** |  |  |  | 8 |  |  |
|  | **16** |  | 2 |  | 3 |  |  |
|  | **17** | 1 | 1 |  |  | 3 |  |
|  | **18** |  | 4 | 3 |  | 4 | 5 |
|  | **19** |  | 4 | 5 |  | 4 | 3 |
|  | **total** | **20** | **30** | **22** | **34** | **27** | **18** |

# Supplementary Table 1: Number of rats used in the experiment divided into the experimental groups.

| Gene | Forward Primer | | Reverse Primer | | Probe |
| --- | --- | --- | --- | --- | --- |
| Total *Bdnf* | AAGTCTGCATTACATTCCTCGA | GTTTTCTGAAAGAGGGACAGTTTAT | | TGTGGTTTGTTGCCGTTGCCAAG | |
| *Clock* | ATCTTTGTCGGCGTTGAGGA | AAAGGTTCGATCACAGCCCA | | CAGAAGCTCAAGAAAGTCCTCG | |
| *Bmal1* | ATCCTGAGCACGGTGAGTTT | \| AAGAGGCGTCGGGACAAAAT \| \| --- \| | | CAACATGCAATGCGATGTCC | |
| *Gad65* | TGAGGGAAATCATTGGCTGG | TCCCCTTTTCCTTGACTTCTG | | TGCCATCTCCAACATGTACGCCA | |
| *Gad67* | ATACTTGGTGTGGCGTAGC | AGGAAAGCAGGTTCTTGGAG | | AAAACTGGGCCTGAAGATCTGTGGT | |
| *36b4* | TCAGTGCCTCACTCCATCAT | AGGAAGGCCTTGACCTTTTC | | TGGATACAAAAGGGTCCTGG | |

**Table 2:** Sequences of forward and reverse primers and probes used in real-time PCR analyses and purchased from Eurofins MWG-Operon.

| Gene | Accession number | Assay ID |
| --- | --- | --- |
| *Bdnf* Long 3’utr | EF125675 | Rn02531967_s1 |
| *Bdnf* Isoform Iv | EF125679 | Rn01484927_m1 |
| *Bdnf* Isoform Vi | EF125680 | Rn01484928_m1 |
| *Ncan* | AF060879 | Rn00581331_m1 |
| *Bcan* | U37142 | Rn00563814_m1 |
| *Hapln1* | HAAF01012309 | Rn00569884_m1 |
| *Sem3a* | HAAF01008759 | Rn00436469_m1 |
| *Nptx2* | S82649.1 | Rn01756377_m1 |

**Table 3:** Probes purchased from Life Technologies which did not disclose the sequence.

| PFC | | | | |
| --- | --- | --- | --- | --- |
| Gene | Experimental group | PND21 | PND35 | PND70 |
| *Total Bdnf* | vehicle | 1.0042 | 0.4645 | 0.6706 |
|  | prenatal-FLX | 0.9904 | 0.5769 | 0.6083 |
|  | postnatal-FLX | 0.8823 | 0.5820 | 0.7067 |
| *Bdnf* long 3’ UTR | vehicle | 0.5420 | 0.2989 | 0.4363 |
|  | prenatal-FLX | 0.5610 | 0.3409 | 0.4176 |
|  | postnatal-FLX | 0.5355 | 0.4071 | 0.3667 |
| *Bdnf* isoform IV | vehicle | 0.5807 | 0.2548 | 0.5499 |
|  | prenatal-FLX | 0.6232 | 0.3956 | 0.5488 |
|  | postnatal-FLX | 0.5401 | 0.6071 | 0.5291 |
| *Bdnf* isoform VI | vehicle | 0.1294 | 0.0837 | 0.1134 |
|  | prenatal-FLX | 0.1789 | 0.1213 | 0.0932 |
|  | postnatal-FLX | 0.1503 | 0.0882 | 0.1000 |
| *Bmal1* | vehicle | 0.3047 | 0.2694 | 0.3102 |
|  | prenatal-FLX | 0.2699 | 0.3337 | 0.2789 |
|  | postnatal-FLX | 0.2478 | 0.2465 | 0.2718 |
| *Clock* | vehicle | 0.6147 | 0.6511 | 0.7231 |
|  | prenatal-FLX | 0.6863 | 0.7021 | 0.6899 |
|  | postnatal-FLX | 0.6554 | 0.6509 | 0.6531 |
| *Gad65* | vehicle | 2.2972 | 1.7317 | 2.1086 |
|  | prenatal-FLX | 2.1030 | 1.9214 | 1.9833 |
|  | postnatal-FLX | 2.2873 | 2.2365 | 2.0277 |
| *Gad67* | vehicle | 0.9290 | 0.8704 | 1.0192 |
|  | prenatal-FLX | 0.8989 | 0.8679 | 1.0413 |
|  | postnatal-FLX | 0.7211 | 0.9751 | 1.0085 |
| *Nptx2* | vehicle | 0.3303 | 0.2160 | 0.2028 |
|  | prenatal-FLX | 0.3157 | 0.2218 | 0.2097 |
|  | postnatal-FLX | 0.2391 | 0.2218 | 0.3182 |
| *Ncan* | vehicle | 1.2942 | 1.3746 | 1.1947 |
|  | prenatal-FLX | 1.6494 | 1.0344 | 1.0366 |
|  | postnatal-FLX | 1.660 | 1.3583 | 1.1294 |
| *Bcan* | vehicle | 0.0693 | 0.1612 | 0.0979 |
|  | prenatal-FLX | 0.0767 | 0.0957 | 0.1239 |
|  | postnatal-FLX | 0.0729 | 0.1238 | 0.1587 |
| *Hapln1* | vehicle | 0.2949 | 0.2699 | 0.2816 |
|  | prenatal-FLX | 0.3191 | 0.3003 | 0.2858 |
|  | postnatal-FLX | 0.3555 | 0.2806 | 0.2538 |
| *Sem3a* | vehicle | 0.5188 | 0.2828 | 0.2330 |
|  | prenatal-FLX | 0.5661 | 0.3805 | 0.2714 |
|  | postnatal-FLX | 0.6493 | 0.3112 | 0.3093 |

**Supplementary Table 4:** total *Bdnf*, *Bdnf* long 3’ UTR, *Bdnf* isoform IV, *Bdnf* isoform VI, *Bmal1, Clock, Gad65, Gad67, Nptx2, Ncan, Bcan, Hapln1, Sem3a,* mRNA levels in the prefrontal cortex of PND21, 35, and 70 male rats exposed to prenatal- or postnatal-FLX. Data are expressed as mean of 2^-Δct^ of independent measures.

| PFC | | | |
| --- | --- | --- | --- |
| Gene | Two-way ANOVA | F (dFn;dFd) | p value |
| *Total Bdnf* | prenatal-FLX | F (1;41) = 0.9740 | p = 0.3303 |
|  | age | F (2;41) = 53.78 | p < 0.0001 |
|  | interaction | F (2;41) = 9.866 | p = 0.0004 |
| *Bdnf* long 3’ UTR | prenatal-FLX | F (1;41) = 0.2530 | p = 0.6180 |
|  | age | F (2;41) = 22.99 | p < 0.0001 |
|  | interaction | F (2;41) = 0.3913 | p = 0.6790 |
| *Bdnf* isoform IV | prenatal-FLX | F (1;41) = 3.507 | p = 0.0692 |
|  | age | F (2;41) = 26.66 | p < 0.0001 |
|  | interaction | F (2;41) = 1.607 | p = 0.2145 |
| *Bdnf* isoform VI | prenatal-FLX | F (1;41) = 9.106 | p = 0.0047 |
|  | age | F (2;41) = 22.06 | p < 0.0001 |
|  | interaction | F (2;41) = 8.626 | p = 0.0009 |
| *Bmal1* | prenatal-FLX | F (1;40) = 0.0037 | p = 0.9518 |
|  | age | F (2;40) = 0.6093 | p = 0.5494 |
|  | interaction | F (2;40) = 9.057 | p = 0.0007 |
| *Clock* | prenatal-FLX | F (1;41) = 1.875 | p = 0.1794 |
|  | age | F (2;41) = 2.294 | p = 0.1155 |
|  | interaction | F (2;41) = 2.209 | p = 0.1244 |
| *Gad65* | prenatal-FLX | F (1;40) = 0.1125 | p = 0.7393 |
|  | age | F (2;40) = 2.758 | p = 0.0772 |
|  | interaction | F (2;40) = 0.7986 | p = 0.4580 |
| *Gad67* | prenatal-FLX | F (1;41) = 0.0055 | p = 0.9412 |
|  | age | F (2;41) = 4.063 | p = 0.0256 |
|  | interaction | F (2;41) = 0.1064 | p = 0.8993 |
| *Nptx2* | prenatal-FLX | F (1;40) = 0.0016 | p = 0.9684 |
|  | age | F (2;40) = 21.39 | p < 0.0001 |
|  | interaction | F (2;40) = 0.1896 | p = 0.8281 |
| *Ncan* | prenatal-FLX | F (1;41) = 0.7890 | p = 0.3803 |
|  | age | F (2;41) = 16.58 | p < 0.0001 |
|  | interaction | F (2;41) = 15.34 | p < 0.0001 |
| *Bcan* | prenatal-FLX | F (1;39) = 2.080 | p = 0.1584 |
|  | age | F (2;39) = 20.44 | p < 0.0001 |
|  | interaction | F (2;39) = 13.52 | p < 0.0001 |
| *Hapln1* | prenatal-FLX | F (1;41) = 2.097 | p = 0.1562 |
|  | age | F (2;41) = 1.296 | p = 0.2860 |
|  | interaction | F (2;41) = 0.3395 | p = 0.7144 |
| *Sem3a* | prenatal-FLX | F (1;41) = 6.916 | p = 0.0125 |
|  | age | F (2;41) = 57.97 | p < 0.0001 |
|  | interaction | F (2;41) = 0.6078 | p = 0.5500 |

**Supplementary Table 5**: Two-way ANOVA analysis of total *Bdnf*, *Bdnf* long 3’ UTR, *Bdnf* isoform IV, *Bdnf* isoform VI, *Bmal1, Clock, Gad65, Gad67, Nptx2, Ncan, Bcan, Hapln1, Sem3a,* mRNA levels in the prefrontal cortex of PND21, 35, and 70 male rats exposed to prenatal-FLX.

| PFC | | | |
| --- | --- | --- | --- |
| Gene | Two-way ANOVA | F (dFn;dFd) | p value |
| *Total Bdnf* | postnatal-FLX | F (1;41) = 13.79 | p = 0.0007 |
|  | age | F (2;41) = 23.79 | p <0.0001 |
|  | interaction | F (2;41) = 9.672 | p = 0.0004 |
| *Bdnf* long 3’ UTR | postnatal-FLX | F (1;41) = 0.098 | p = 0.7561 |
|  | age | F (2.41) = 10.10 | p = 0.0003 |
|  | interaction | F (2;41) = 2.438 | p = 0.1017 |
| *Bdnf* isoform IV | postnatal-FLX | F (1;41) = 4.859 | p = 0.0340 |
|  | age | F (2;41) = 3.303 | p = 0.0482 |
|  | interaction | F (2;41) = 8.476 | p = 0.0010 |
| *Bdnf* isoform VI | postnatal-FLX | F (1;41) = 0.2360 | p = 0.6300 |
|  | age | F (2;41) = 13.93 | p < 0.0001 |
|  | interaction | F (2;41) = 1.458 | p = 0.2460 |
| *Bmal1* | postnatal-FLX | F (1;40) = 11.24 | p = 0.0019 |
|  | age | F (2;40) = 2.752 | p = 0.0776 |
|  | interaction | F (2;40) = 0.6770 | p = 0.5147 |
| *Clock* | postnatal-FLX | F (1;41) = 0.1467 | p = 0.7040 |
|  | age | F (2;41) = 1.551 | p = 0.2258 |
|  | interaction | F (2;41) = 1.628 | p = 0.2105 |
| *Gad65* | postnatal-FLX | F (1;41) = 0.5465 | p = 0.4646 |
|  | age | F (2;41) = 0.9195 | p = 0.4079 |
|  | interaction | F (2;41) = 0.9975 | p = 0.3788 |
| *Gad67* | postnatal-FLX | F (1;41) = 0.3960 | p = 0.5331 |
|  | age | F (2;41) = 3.250 | p = 0.0504 |
|  | interaction | F (2;41) = 2.174 | p = 0.1285 |
| *Nptx2* | postnatal-FLX | F (1;41) = 0.5463 | p = 0.4646 |
|  | age | F (2;41) = 2.699 | p = 0.0809 |
|  | interaction | F (2;41) = 8.625 | p = 0.0009 |
| *Ncan* | postnatal-FLX | F (1;41) = 3.002 | p = 0.0917 |
|  | age | F (2;41) = 11.58 | p = 0.0001 |
|  | interaction | F (2;41) = 5.948 | p = 0.0059 |
| *Bcan* | postnatal-FLX | F (1;39) = 0.6559 | p = 0.4236 |
|  | age | F (2;39) = 14.82 | p < 0.0001 |
|  | interaction | F (2;39) = 6.698 | p = 0.0035 |
| *Hapln1* | postnatal-FLX | F (1;41) = 1.031 | p = 0.3168 |
|  | age | F (2;41) = 6.118 | p = 0.0052 |
|  | interaction | F (2;41) = 3.185 | p = 0.0533 |
| *Sem3a* | postnatal-FLX | F (1;41) = 7.806 | p = 0.0083 |
|  | age | F (2;41) = 48.83 | p < 0.0001 |
|  | interaction | F (2;41) = 1.055 | p = 0.3587 |

**Supplementary Table 6**: Two-way ANOVA analysis of total *Bdnf*, *Bdnf* long 3’ UTR, *Bdnf* isoform IV, *Bdnf* isoform VI, *Bmal1, Clock, Gad65, Gad67, Nptx2, Ncan, Bcan, Hapln1, Sem3a,* mRNA levels in the prefrontal cortex of PND21, 35, and 70 male rats exposed to postnatal-FLX.

| dHip | | | | |
| --- | --- | --- | --- | --- |
| Gene | Experimental group | PND21 | PND35 | PND70 |
| *Total Bdnf* | vehicle | 0.6754 | 0.4454 | 0.8901 |
|  | prenatal-FLX | 0.5838 | 0.6629 | 0.6648 |
|  | postnatal-FLX | 0.6317 | 0.5325 | 0.6554 |
| *Bdnf* long 3’ UTR | vehicle | 0.5471 | 0.4028 | 0.5673 |
|  | prenatal-FLX | 0.4447 | 0.3807 | 0.3943 |
|  | postnatal-FLX | 0.4720 | 0.3498 | 0.4630 |
| *Bdnf* isoform IV | vehicle | 0.1372 | 0.1236 | 0.1712 |
|  | prenatal-FLX | 0.1428 | 0.1044 | 0.1180 |
|  | postnatal-FLX | 0.1403 | 0.1370 | 0.1363 |
| *Bdnf* isoform VI | vehicle | 0.1520 | 0.0902 | 0.1622 |
|  | prenatal-FLX | 0.1696 | 0.1368 | 0.1406 |
|  | postnatal-FLX | 0.1549 | 0.1128 | 0.1615 |
| *Bmal1* | vehicle | 0.3595 | 0.3069 | 0.2550 |
|  | prenatal-FLX | 0.2548 | 0.2348 | 0.2084 |
|  | postnatal-FLX | 0.2110 | 0.2394 | 0.2639 |
| *Clock* | vehicle | 0.4445 | 0.5365 | 0.4381 |
|  | prenatal-FLX | 0.5575 | 0.4297 | 0.4081 |
|  | postnatal-FLX | 0.4162 | 0.3682 | 0.4312 |
| *Gad65* | vehicle | 1.5683 | 1.5634 | 1.5779 |
|  | prenatal-FLX | 1.5459 | 1.4520 | 1.0531 |
|  | postnatal-FLX | 1.5161 | 1.2367 | 1.2411 |
| *Gad67* | vehicle | 1.3769 | 1.3735 | 1.2700 |
|  | prenatal-FLX | 1.4408 | 1.2308 | 0.9688 |
|  | postnatal-FLX | 1.1578 | 1.1727 | 1.1606 |
| *Nptx2* | vehicle | 0.1332 | 0.1832 | 0.1133 |
|  | prenatal-FLX | 0.1096 | 0.1064 | 0.1058 |
|  | postnatal-FLX | 0.0977 | 0.2024 | 0.1114 |
| *Ncan* | vehicle | 0.9556 | 0.9050 | 0.9454 |
|  | prenatal-FLX | 1.4220 | 0.9358 | 0.8618 |
|  | postnatal-FLX | 1.2976 | 0.9752 | 0.8410 |
| *Bcan* | vehicle | 0.0413 | 0.1678 | 0.0825 |
|  | prenatal-FLX | 0.0520 | 0.0840 | 0.1068 |
|  | postnatal-FLX | 0.0442 | 0.1048 | 0.0735 |
| *Hapln1* | vehicle | 0.2262 | 0.1814 | 0.1824 |
|  | prenatal-FLX | 0.1914 | 0.1748 | 0.1674 |
|  | postnatal-FLX | 0.1883 | 0.1651 | 0.1475 |
| *Sem3a* | vehicle | 0.0859 | 0.1463 | 0.0318 |
|  | prenatal-FLX | 0.0851 | 0.0392 | 0.0286 |
|  | postnatal-FLX | 0.0746 | 0.0577 | 0.0283 |

**Supplementary Table 7:** total *Bdnf*, *Bdnf* long 3’ UTR, *Bdnf* isoform IV, *Bdnf* isoform VI, *Bmal1, Clock, Gad65, Gad67, Nptx2, Ncan, Bcan, Hapln1, Sem3a,* mRNA levels in the dorsal hippocampus of PND21, 35, and 70 male rats exposed to prenatal- or postnatal-FLX. Data are expressed as mean of 2^-Δct^ of independent measures.

| dHip | | | |
| --- | --- | --- | --- |
| Gene | Two-way ANOVA | F (dFn;dFd) | p value |
| *Total Bdnf* | prenatal-FLX | F (1;41) = 1.046 | p = 0.3132 |
|  | age | F (2;41) = 16.09 | p < 0.0001 |
|  | interaction | F (2;41) = 15.78 | p < 0.0001 |
| *Bdnf* long 3’ UTR | prenatal-FLX | F (1;41) = 7.821 | p = 0.0082 |
|  | age | F (2;41) = 3.258 | p = 0.0501 |
|  | interaction | F (2;41) = 1.460 | p = 0.2457 |
| *Bdnf* isoform IV | prenatal-FLX | F (1;41) = 6.364 | p = 0.0162 |
|  | age | F (2;41) = 4.476 | p = 0.0184 |
|  | interaction | F (2;41) = 3.859 | p = 0.0303 |
| *Bdnf* isoform VI | prenatal-FLX | F (1;41) = 3.253 | p = 0.0797 |
|  | age | F (2;41) = 13.20 | p < 0.0001 |
|  | interaction | F (2;41) = 6.151 | p = 0.0050 |
| *Bmal1* | prenatal-FLX | F (1;41) = 56.17 | p < 0.0001 |
|  | age | F (2;41) = 19.96 | p < 0.0001 |
|  | interaction | F (2;41) = 2.984 | p = 0.0632 |
| *Clock* | prenatal-FLX | F (1;41) = 0.2154 | p = 0.6454 |
|  | age | F (2;41) = 7.802 | p = 0.0015 |
|  | interaction | F (2;41) = 14.41 | p < 0.0001 |
| *Gad65* | prenatal-FLX | F (1;41) = 6.962 | p = 0.0122 |
|  | age | F (2;41) = 3.204 | p = 0.0524 |
|  | interaction | F (2;41) = 3.528 | p = 0.0399 |
| *Gad67* | prenatal-FLX | F (1;41) = 2.537 | p = 0.1200 |
|  | age | F (2;41) = 4.666 | p = 0.0158 |
|  | interaction | F (2;41) = 1.841 | p = 0.1733 |
| *Nptx2* | prenatal-FLX | F (1;41) = 28.36 | p < 0.0001 |
|  | age | F (2;41) = 9.064 | p = 0.0006 |
|  | interaction | F (2;41) = 9.243 | p = 0.0006 |
| *Ncan* | prenatal-FLX | F (1;41) = 16.98 | p = 0.0002 |
|  | age | F (2;41) = 31.68 | p < 0.0001 |
|  | interaction | F (2;41) = 26.15 | p < 0.0001 |
| *Bcan* | prenatal-FLX | F (1;40) = 1.120 | p = 0.2972 |
|  | age | F (2;40) = 9.051 | p = 0.0007 |
|  | interaction | F (2;40) = 4.604 | p = 0.01681 |
| *Hapln1* | prenatal-FLX | F (1;41) = 7.982 | p = 0.0077 |
|  | age | F (2;41) = 10.94 | p = 0.0002 |
|  | interaction | F (2;41) = 1.604 | p = 0.2151 |
| *Sem3a* | prenatal-FLX | F (1;41) = 11.19 | p = 0.0019 |
|  | age | F (2;41) = 12.65 | p < 0.0001 |
|  | interaction | F (2;41) = 9.642 | p = 0.0004 |

**Supplementary Table 8**: Two-way ANOVA analysis of total *Bdnf*, *Bdnf* long 3’ UTR, *Bdnf* isoform IV, *Bdnf* isoform VI, *Bmal1, Clock, Gad65, Gad67, Nptx2, Ncan, Bcan, Hapln1, Sem3a,* mRNA levels in the dorsal hippocampus of PND21, 35, and 70 male rats exposed to prenatal-FLX.

| dHip | | | |
| --- | --- | --- | --- |
| Gene | Two-way ANOVA | F (dFn;dFd) | p value |
| *Total Bdnf* | postnatal-FLX | F (1;41) = 4.315 | p = 0.0450 |
|  | age | F (2;41) = 30.16 | p < 0.0001 |
|  | interaction | F (2;41) = 9.808 | p = 0.0004 |
| *Bdnf* long 3’ UTR | postnatal-FLX | F (1;41) = 5.017 | p = 0.0314 |
|  | age | F (2;41) = 6.977 | p = 0.0027 |
|  | interaction | F (2;41) = 0.1953 | p = 0.8234 |
| *Bdnf* isoform IV | postnatal-FLX | F (1;41) = 0.3647 | p = 0.5497 |
|  | age | F (2;41) = 1.917 | p = 0.1618 |
|  | interaction | F (2;41) = 2.207 | p = 0.1247 |
| *Bdnf* isoform VI | postnatal-FLX | F (1;41) = 0.9036 | p = 0.3482 |
|  | age | F (2;41) = 19.32 | p < 0.0001 |
|  | interaction | F (2;41) = 0.7137 | p = 0.4966 |
| *Bmal1* | postnatal-FLX | F (1;41) = 30.59 | p < 0.0001 |
|  | age | F (2;41) = 1.425 | p = 0.2537 |
|  | interaction | F (2;41) = 13.21 | p < 0.0001 |
| *Clock* | postnatal-FLX | F (1;41) = 16.04 | p = 0.0003 |
|  | age | F (2;41) = 0.6225 | p = 0.5423 |
|  | interaction | F (2;41) = 9.121 | p = 0.0006 |
| *Gad65* | postnatal-FLX | F (1;41) = 11.54 | p = 0.0017 |
|  | age | F (2;41) = 1.621 | p = 0.2118 |
|  | interaction | F (2;41) = 1.681 | p = 0.2005 |
| *Gad67* | postnatal-FLX | F (1;41) = 8.541 | p = 0.0060 |
|  | age | F (2;41) = 0.3909 | p = 0.6793 |
|  | interaction | F (2;41) = 0.3275 | p = 0.7228 |
| *Nptx2* | postnatal-FLX | F (1;41) = 0.1616 | p = 0.6901 |
|  | age | F (2;41) = 12.34 | p < 0.0001 |
|  | interaction | F (2;41) = 1.059 | p = 0.3572 |
| *Ncan* | postnatal-FLX | F (1;41) = 8.448 | p = 0.0062 |
|  | age | F (2;41) = 15.73 | p < 0.0001 |
|  | interaction | F (2.41) = 13.32 | p < 0.0001 |
| *Bcan* | postnatal-FLX | F (1;40) = 1.942 | p = 0.1723 |
|  | age | F (2;40) = 10.38 | p = 0.0003 |
|  | interaction | F (2;40) = 1.463 | p = 0.2453 |
| *Hapln1* | postnatal-FLX | F (1;41) = 14.34 | p = 0.0006 |
|  | age | F (2;41) = 10.51 | p = 0.0003 |
|  | interaction | F (2;41) = 0.7380 | p = 0.4852 |
| *Sem3a* | postnatal-FLX | F (1;40) = 7.487 | p = 0.0097 |
|  | age | F (2;40) = 12.07 | p = 0.0001 |
|  | interaction | F (2;40) = 4.636 | p = 0.0164 |

**Supplementary Table 9**: Two-way ANOVA analysis of total *Bdnf*, *Bdnf* long 3’ UTR, *Bdnf* isoform IV, *Bdnf* isoform VI, *Bmal1, Clock, Gad65, Gad67, Nptx2, Ncan, Bcan, Hapln1, Sem3a,* mRNA levels in the dorsal hippocampus of PND21, 35, and 70 male rats exposed to postnatal-FLX.

| PFC | | | | |
| --- | --- | --- | --- | --- |
| Gene | Experimental group | PND21 | PND35 | PND70 |
| *Total Bdnf* | vehicle | 0.9355 | 0.4343 | 0.5087 |
|  | prenatal-FLX | 0.9092 | 0.7424 | 0.5679 |
|  | postnatal-FLX | 0.8099 | 0.7382 | 0.4592 |
| *Bdnf* long 3’ UTR | vehicle | 0.5478 | 0.3515 | 0.2697 |
|  | prenatal-FLX | 0.5386 | 0.3740 | 0.4541 |
|  | postnatal-FLX | 0.4082 | 0.4822 | 0.2578 |
| *Bdnf* isoform IV | vehicle | 0.4497 | 0.2309 | 0.2374 |
|  | prenatal-FLX | 0.3854 | 0.2389 | 0.3341 |
|  | postnatal-FLX | 0.3790 | 0.4261 | 0.1854 |
| *Bdnf* isoform VI | vehicle | 0.1429 | 0.0663 | 0.0871 |
|  | prenatal-FLX | 0.1452 | 0.1016 | 0.1227 |
|  | postnatal-FLX | 0.1203 | 0.1003 | 0.0975 |
| *Bmal1* | vehicle | 0.2655 | 0.2317 | 0.2322 |
|  | prenatal-FLX | 0.2624 | 0.2801 | 0.2962 |
|  | postnatal-FLX | 0.2375 | 0.2684 | 0.2456 |
| *Clock* | vehicle | 0.7037 | 0.6514 | 0.6525 |
|  | prenatal-FLX | 0.6892 | 0.5796 | 0.7157 |
|  | postnatal-FLX | 0.6341 | 0.6251 | 0.6137 |
| *Gad65* | vehicle | 1.9364 | 2.7070 | 2.0631 |
|  | prenatal-FLX | 2.1684 | 2.0149 | 1.8150 |
|  | postnatal-FLX | 2.1291 | 2.4138 | 2.3848 |
| *Gad67* | vehicle | 0.8163 | 1.2120 | 1.0472 |
|  | prenatal-FLX | 0.9481 | 0.9476 | 0.8260 |
|  | postnatal-FLX | 0.8520 | 1.1938 | 1.0448 |
| *Nptx2* | vehicle | 0.2979 | 0.2465 | 0.2116 |
|  | prenatal-FLX | 0.3079 | 0.2727 | 0.2882 |
|  | postnatal-FLX | 0.2253 | 0.2787 | 0.2789 |
| *Ncan* | vehicle | 0.5918 | 0.4131 | 0.4816 |
|  | prenatal-FLX | 0.5759 | 0.4069 | 0.5320 |
|  | postnatal-FLX | 0.5735 | 0.5405 | 0.4886 |
| *Bcan* | vehicle | 0.0684 | 0.1157 | 0.1327 |
|  | prenatal-FLX | 0.0739 | 0.0769 | 0.1651 |
|  | postnatal-FLX | 0.0565 | 0.1567 | 0.1878 |
| *Hapln1* | vehicle | 0.3139 | 0.2652 | 0.2423 |
|  | prenatal-FLX | 0.3194 | 0.2682 | 0.2934 |
|  | postnatal-FLX | 0.3506 | 0.2811 | 0.2659 |
| *Sem3a* | vehicle | 0.3621 | 0.3254 | 0.1745 |
|  | prenatal-FLX | 0.4059 | 0.3685 | 0.2278 |
|  | postnatal-FLX | 0.3731 | 0.2640 | 0.2652 |

**Supplementary Table 10:** total *Bdnf*, *Bdnf* long 3’ UTR, *Bdnf* isoform IV, *Bdnf* isoform VI, *Bmal1, Clock, Gad65, Gad67, Nptx2, Ncan, Bcan, Hapln1, Sem3a,* mRNA levels in the prefrontal cortex of PND21, 35, and 70 female rats exposed to prenatal- or postnatal-FLX. Data are expressed as mean of 2^-Δct^ of independent measures.

| PFC | | | |
| --- | --- | --- | --- |
| Gene | Two-way ANOVA | F (dFn;dFd) | p value |
| *Total Bdnf* | prenatal-FLX | F (1;37) = 2.320 | p = 0.1376 |
|  | age | F (2;37) = 9.518 | p = 0.0006 |
|  | interaction | F (2;37) = 1.271 | p = 0.2944 |
| *Bdnf* long 3’ UTR | prenatal-FLX | F (1;37) = 5.596 | p = 0.0242 |
|  | age | F (2;37) = 22.86 | p < 0.0001 |
|  | interaction | F (2;37) = 4.600 | p = 0.0175 |
| *Bdnf* isoform IV | prenatal-FLX | F (1;37) = 0.1326 | p = 0.7182 |
|  | age | F (2;37) = 10.37 | p = 0.0003 |
|  | interaction | F (2;37) = 1.759 | p = 0.1884 |
| *Bdnf* isoform VI | prenatal-FLX | F (1;37) = 8.172 | p = 0.0074 |
|  | age | F (2;37) = 20.02 | p < 0.0001 |
|  | interaction | F (2;37) = 2.045 | p = 0.1459 |
| *Bmal1* | prenatal-FLX | F (1;37) = 5.562 | p = 0.0246 |
|  | age | F (2;37) = 0.1164 | p = 0.8905 |
|  | interaction | F (2;37) = 2.067 | p = 0.1431 |
| *Clock* | prenatal-FLX | F (1;37) = 0.1003 | p = 0.7535 |
|  | age | F (2;37) = 4.245 | p = 0.0232 |
|  | interaction | F (2;37) = 2.203 | p = 0.1270 |
| *Gad65* | prenatal-FLX | F (1;37) = 1.699 | p = 0.2017 |
|  | age | F (2;37) = 1.689 | p = 0.2007 |
|  | interaction | F (2;37) = 2.463 | p = 0.1011 |
| *Gad67* | prenatal-FLX | F (1;37) = 4.537 | p = 0.0410 |
|  | age | F (2;37) = 4.702 | p = 0.0162 |
|  | interaction | F (2;37) = 6.262 | p = 0.0051 |
| *Nptx2* | prenatal-FLX | F (1;36) = 3.482 | p = 0.0715 |
|  | age | F (2;36) = 3.231 | p = 0.0531 |
|  | interaction | F (2;36) = 1.048 | p = 0.3628 |
| *Ncan* | prenatal-FLX | F (1;37) = 0.0477 | p = 0.8285 |
|  | age | F (2;37) = 6.009 | p = 0.0061 |
|  | interaction | F (2;37) = 0.2265 | p = 0.7986 |
| *Bcan* | prenatal-FLX | F (1;34) = 0.0613 | p = 0.8061 |
|  | age | F (2;34) = 8.783 | p = 0.0010 |
|  | interaction | F (2;34) = 1.355 | p = 0.2739 |
| *Hapln1* | prenatal-FLX | F (1;36) = 3.584 | p = 0.0677 |
|  | age | F (2;36) = 11.90 | p = 0.0001 |
|  | interaction | F (2;36) = 2.069 | p = 0.1434 |
| *Sem3a* | prenatal-FLX | F (1;37) = 2.422 | p = 0.1294 |
|  | age | F (2;37) = 13.95 | p < 0.0001 |
|  | interaction | F (2;37) = 0.0110 | p = 0.9890 |

**Supplementary Table 11**: Two-way ANOVA analysis of total *Bdnf*, *Bdnf* long 3’ UTR, *Bdnf* isoform IV, *Bdnf* isoform VI, *Bmal1, Clock, Gad65, Gad67, Nptx2, Ncan, Bcan, Hapln1, Sem3a,* mRNA levels in the prefrontal cortex of PND21, 35, and 70 female rats exposed to prenatal-FLX.

| PFC | | | |
| --- | --- | --- | --- |
| Gene | Two-way ANOVA | F (dFn; dFd) | p value |
| *Total Bdnf* | postnatal-FLX | F (1;52) = 0.8053 | p = 0.3741 |
|  | age | F (2;52) = 15.77 | p < 0.0001 |
|  | interaction | F (2;52) = 4.173 | p = 0.0215 |
| *Bdnf* long 3’ UTR | postnatal-FLX | F (1;52) = 0.0836 | p = 0.7737 |
|  | age | F (2;52) = 29.02 | p < 0.0001 |
|  | interaction | F (2.52) = 12.12 | p < 0.0001 |
| *Bdnf* isoform IV | postnatal-FLX | F (1;52) = 0.6490 | p = 0.4245 |
|  | age | F (2;52) = 16.62 | p < 0.0001 |
|  | interaction | F (2;52) = 8.029 | p = 0.0010 |
| *Bdnf* isoform VI | postnatal-FLX | F (1;52) = 1.253 | p = 0.2687 |
|  | age | F (2;52) = 25.44 | p < 0.0001 |
|  | interaction | F (2;52) = 7.517 | p = 0.0015 |
| *Bmal1* | postnatal-FLX | F (1;52) = 0.4937 | p = 0.4858 |
|  | age | F (2;52) = 0.5562 | p = 0.5771 |
|  | interaction | F (2;52) = 3.901 | p = 0.0271 |
| *Clock* | postnatal-FLX | F (1;52) = 5.224 | p = 0.0268 |
|  | age | F (2;52) = 1.561 | p = 0.2206 |
|  | interaction | F (2;52) = 0.5141 | p = 0.6013 |
| *Gad65* | postnatal-FLX | F (1;52) = 0.1627 | p = 0.6885 |
|  | age | F (2;52) = 3.113 | p = 0.0537 |
|  | interaction | F (2;52) = 0.9410 | p = 0.3975 |
| *Gad67* | postnatal-FLX | F (1;52) = 0.0062 | p = 0.9376 |
|  | age | F (2;52) = 12.83 | p < 0.0001 |
|  | interaction | F (2;52) = 0.0739 | p = 0.9289 |
| *Nptx2* | postnatal-FLX | F (1;50) = 0.2725 | p = 0.6042 |
|  | age | F (2;50) = 0.4055 | p = 0.6691 |
|  | interaction | F (2;50) = 7.327 | p = 0.0018 |
| *Ncan* | postnatal-FLX | F (1;51) = 1.258 | p = 0.2678 |
|  | age | F (2;51) = 4.704 | p = 0.0138 |
|  | interaction | F (2;51) = 1.723 | p = 0.1898 |
| *Bcan* | postnatal-FLX | F (1;49) = 2.715 | p = 0.1065 |
|  | age | F (2;49) = 14.27 | p < 0.0001 |
|  | interaction | F (2;49) = 1.722 | p = 0.1905 |
| *Hapln1* | postnatal-FLX | F (1;51) = 7.298 | p = 0.0096 |
|  | age | F (2;51) = 29.33 | p < 0.0001 |
|  | interaction | F (2;51) = 0.4847 | p = 0.6190 |
| *Sem3a* | postnatal-FLX | F (1;51) = 0.6829 | p = 0.4129 |
|  | age | F (2;51) = 31.04 | p < 0.0001 |
|  | interaction | F (2.51) = 6.127 | p = 0.0044 |

**Supplementary Table 12**: Two-way ANOVA analysis of total *Bdnf*, *Bdnf* long 3’ UTR, *Bdnf* isoform IV, *Bdnf* isoform VI, *Bmal1, Clock, Gad65, Gad67, Nptx2, Ncan, Bcan, Hapln1, Sem3a* mRNA levels in the prefrontal cortex of PND21, 35, and 70 female rats exposed to postnatal-FLX.

| dHip | | | | |
| --- | --- | --- | --- | --- |
| Gene | Experimental group | PND21 | PND35 | PND70 |
| *Total Bdnf* | vehicle | 0.5113 | 0.4428 | 0.5875 |
|  | prenatal-FLX | 0.4323 | 0.3873 | 0.3328 |
|  | postnatal-FLX | 0.3476 | 0.4359 | 0.5862 |
| *Bdnf* long 3’ UTR | vehicle | 0.4177 | 0.3427 | 0.4236 |
|  | prenatal-FLX | 0.4221 | 0.3615 | 0.3260 |
|  | postnatal-FLX | 0.4189 | 0.3874 | 0.4637 |
| *Bdnf* isoform IV | vehicle | 0.1309 | 0.1060 | 0.1193 |
|  | prenatal-FLX | 0.1217 | 0.0849 | 0.0860 |
|  | postnatal-FLX | 0.1115 | 0.1060 | 0.1285 |
| *Bdnf* isoform VI | vehicle | 0.1782 | 0.1168 | 0.1687 |
|  | prenatal-FLX | 0.1547 | 0.1276 | 0.1414 |
|  | postnatal-FLX | 0.1532 | 0.1755 | 0.1709 |
| *Bmal1* | vehicle | 0.3956 | 0.3275 | 0.3301 |
|  | prenatal-FLX | 0.3577 | 0.3776 | 0.3229 |
|  | postnatal-FLX | 0.3260 | 0.3397 | 0.3716 |
| *Clock* | vehicle | 0.4822 | 0.5168 | 0.4573 |
|  | prenatal-FLX | 0.4704 | 0.5313 | 0.4831 |
|  | postnatal-FLX | 0.4554 | 0.5453 | 0.6110 |
| *Gad65* | vehicle | 1.6685 | 1.1807 | 1.2645 |
|  | prenatal-FLX | 1.4050 | 0.9581 | 1.3646 |
|  | postnatal-FLX | 1.6178 | 1.2315 | 1.3593 |
| *Gad67* | vehicle | 0.9437 | 0.7376 | 0.6641 |
|  | prenatal-FLX | 0.8467 | 0.6768 | 0.7417 |
|  | postnatal-FLX | 0.8083 | 0.7464 | 0.6970 |
| *Nptx2* | vehicle | 0.1021 | 0.1161 | 0.1275 |
|  | prenatal-FLX | 0.0864 | 0.0894 | 0.0891 |
|  | postnatal-FLX | 0.0911 | 0.1534 | 0.1464 |
| *Ncan* | vehicle | 1.1085 | 0.9499 | 0.7008 |
|  | prenatal-FLX | 1.2201 | 0.8497 | 0.8196 |
|  | postnatal-FLX | 1.0102 | 0.9193 | 0.8351 |
| *Bcan* | vehicle | 0.0592 | 0.2060 | 0.0866 |
|  | prenatal-FLX | 0.0592 | 0.0844 | 0.0819 |
|  | postnatal-FLX | 0.0457 | 0.0964 | 0.0733 |
| *Hapln1* | vehicle | 0.0900 | 0.0617 | 0.0594 |
|  | prenatal-FLX | 0.0899 | 0.0649 | 0.0557 |
|  | postnatal-FLX | 0.0830 | 0.0732 | 0.0580 |
| *Sem3a* | vehicle | 0.0600 | 0.0301 | 0.0200 |
|  | prenatal-FLX | 0.0728 | 0.0306 | 0.0323 |
|  | postnatal-FLX | 0.0535 | 0.0382 | 0.0314 |

**Supplementary Table 13:** total *Bdnf*. *Bdnf* long 3’ UTR. *Bdnf* isoform IV. *Bdnf* isoform VI. *Bmal1, Clock, Gad65, Gad67, Nptx2, Ncan, Bcan, Hapln1, Sem3a,* mRNA levels in the dorsal hippocampus of PND21, 35, and 70 female rats exposed to prenatal- or postnatal-FLX. Data are expressed as mean of 2^-Δct^ of independent measures.

| dHip | | | |
| --- | --- | --- | --- |
| Gene | Two-way ANOVA | F (dFn;dFd) | p value |
| *Total Bdnf* | prenatal-FLX | F (1;36) = 18.87 | p=0.0001 |
|  | age | F (2;36) = 1.280 | p=0.2925 |
|  | interaction | F (2;36) = 4.221 | p=0.0239 |
| *Bdnf* long 3’ UTR | prenatal-FLX | F (1;36) = 1.655 | p=0.2078 |
|  | age | F (2;36) = 5.038 | p=0.0127 |
|  | interaction | F (2;36) = 3.447 | p=0.0445 |
| *Bdnf* isoform IV | prenatal-FLX | F (1;37) = 11.05 | p=0.0022 |
|  | age | F (2;37) = 10.24 | p=0.0004 |
|  | interaction | F (2;37) = 1.326 | p=0.2797 |
| *Bdnf* isoform VI | prenatal-FLX | F (1;37) = 5.201 | p=0.0294 |
|  | age | F (2;37) = 21.15 | p<0.0001 |
|  | interaction | F (2;37) = 3.900 | p=0.0305 |
| *Bmal1* | prenatal-FLX | F (1;36) = 0.0189 | p=0.8915 |
|  | age | F (2;36) = 6.920 | p=0.0033 |
|  | interaction | F (2;36) = 4.642 | p=0.0172 |
| *Clock* | prenatal-FLX | F (1;37) = 0.1289 | p=0.7219 |
|  | age | F (2;37) = 1.497 | p=0.2390 |
|  | interaction | F (2;37) = 0.2092 | p=0.8123 |
| *Gad65* | prenatal-FLX | F (1;37) = 1.802 | p=0.1889 |
|  | age | F (2;37) = 8.869 | p=0.0009 |
|  | interaction | F (2;37) = 1.403 | p=0.2605 |
| *Gad67* | prenatal-FLX | F (1;37) = 0.5906 | p=0.4478 |
|  | age | F (2;37) = 16.18 | p<0.0001 |
|  | interaction | F (2;37) = 2.359 | p=0.1107 |
| *Nptx2* | prenatal-FLX | F (1;36) = 32.11 | p<0.0001 |
|  | age | F (2;36) = 3.704 | p=0.0361 |
|  | interaction | F (2;36) = 2.308 | p=0.1163 |
| *Ncan* | prenatal-FLX | F (1;37) = 1.787 | p=0.1907 |
|  | age | F (2;37) = 62.70 | p<0.0001 |
|  | interaction | F (2;37) = 4.553 | p=0.0182 |
| *Bcan* | prenatal-FLX | F (1;36) = 5.290 | p=0.0283 |
|  | age | F (2;36) = 7.730 | p=0.0019 |
|  | interaction | F (2;36) = 4.218 | p=0.0240 |
| *Hapln1* | prenatal-FLX | F (1;37) = 0.0033 | p=0.9547 |
|  | age | F (2;37) = 36.85 | p<0.0001 |
|  | interaction | F (2;37) = 0.2569 | p=0.7750 |
| *Sem3a* | prenatal-FLX | F (1;36) = 4.417 | p=0.0438 |
|  | age | F (2;36) = 50.40 | p<0.0001 |
|  | interaction | F (2;36) = 0.8808 | p=0.4246 |

**Supplementary Table 14**: Two-way ANOVA analysis of total *Bdnf*, *Bdnf* long 3’ UTR, *Bdnf* isoform IV, *Bdnf* isoform VI, *Bmal1, Clock, Gad65, Gad67, Nptx2, Ncan, Bcan, Hapln1, Sem3a* mRNA levels in the dorsal hippocampus of PND21, 35, and 70 female rats exposed to prenatal-FLX.

| dHip | | | |
| --- | --- | --- | --- |
| Gene | Two-way ANOVA | F (dFn;dFd) | p value |
| *Total Bdnf* | postnatal-FLX | F (1;52) = 6.305 | p=0.0155 |
|  | age | F (2;52) = 19.50 | p<0.0001 |
|  | interaction | F (2;52) = 6.660 | p=0.0028 |
| *Bdnf* long 3’ UTR | postnatal-FLX | F (1;53) = 2.378 | p=0.1296 |
|  | age | F (2;53) = 5.378 | p=0.0078 |
|  | interaction | F (2;53) = 0.6629 | p=0.5200 |
| *Bdnf* isoform IV | postnatal-FLX | F (1;53) = 0.7574 | p=0.3885 |
|  | age | F (2;53) = 3.530 | p=0.0371 |
|  | interaction | F (2;53) = 3.775 | p=0.0300 |
| *Bdnf* isoform VI | postnatal-FLX | F (1;53) = 3.779 | p=0.0578 |
|  | age | F (2;53) = 5.048 | p=0.0102 |
|  | interaction | F (2;53) = 16.90 | p<0.0001 |
| *Bmal1* | postnatal-FLX | F (1;52) = 0.2417 | p=0.6252 |
|  | age | F (2;52) = 2.300 | p=0.1114 |
|  | interaction | F (2;52) = 11.51 | p<0.0001 |
| *Clock* | postnatal-FLX | F (1;53) = 8.836 | p=0.0046 |
|  | age | F (2;53) = 7.228 | p=0.0018 |
|  | interaction | F (2;53) = 9.825 | p=0.0003 |
| *Gad65* | postnatal-FLX | F (1;53) = 0.1461 | p=0.7039 |
|  | age | F (2;53) = 12.05 | p<0.0001 |
|  | interaction | F (2;53) = 0.3194 | p=0.7281 |
| *Gad67* | postnatal-FLX | F (1;53) = 1.220 | p=0.2749 |
|  | age | F (2;53) = 19.54 | p<0.0001 |
|  | interaction | F (2;53) = 4.148 | p=0.0218 |
| *Nptx2* | postnatal-FLX | F (1;52) = 8.788 | p=0.0048 |
|  | age | F (2;52) = 32.47 | p<0.0001 |
|  | interaction | F (2;52) = 8.823 | p=0.0006 |
| *Ncan* | postnatal-FLX | F (1;52) = 0.0031 | p=0.9557 |
|  | age | F (2;52) = 30.22 | p<0.0001 |
|  | interaction | F (2;52) = 4.787 | p=0.0128 |
| *Bcan* | postnatal-FLX | F (1;51) = 14.45 | p=0.0004 |
|  | age | F (2;51) = 24.15 | p<0.0001 |
|  | interaction | F (2;51) = 6.458 | p=0.0034 |
| *Hapln1* | postnatal-FLX | F (1;53) = 0.0702 | p=0.7921 |
|  | age | F (2;53) = 20.12 | p<0.0001 |
|  | interaction | F (2;53) = 1.976 | p=0.1497 |
| *Sem3a* | postnatal-FLX | F (1;52) = 0.7036 | p=0.4058 |
|  | age | F (2;52) = 15.16 | p<0.0001 |
|  | interaction | F (2;52) = 1.340 | p=0.2716 |

**Supplementary Table 15**: Two-way ANOVA analysis of total *Bdnf*, *Bdnf* long 3’ UTR, *Bdnf* isoform IV, *Bdnf* isoform VI, *Bmal1, Clock, Gad65, Gad67, Nptx2, Ncan, Bcan, Hapln1, Sem3a,* mRNA levels in the dorsal hippocampus of PND21, 35, and 70 female rats exposed to postnatal-FLX.
